# Endocrine Dysfunction in Female *FMR1* Premutation Carriers: Characteristics and Association with Ill Health

**DOI:** 10.3390/genes7110101

**Published:** 2016-11-18

**Authors:** Sonya Campbell, Sarah E. A. Eley, Andrew G. McKechanie, Andrew C. Stanfield

**Affiliations:** The Patrick Wild Centre, The University of Edinburgh, Royal Edinburgh Hospital, Edinburgh EH10 5HF, UK; s.eley@ed.ac.uk (S.E.A.E.); andrew.mckechanie@ed.ac.uk (A.G.M.); andrew.stanfield@ed.ac.uk (A.C.S.)

**Keywords:** fragile X, premutation, endocrine, health, premature ovarian insufficiency

## Abstract

Female *FMR1* premutation carriers (PMC) have been suggested to be at greater risk of ill health, in particular endocrine dysfunction, compared to the general population. We set out to review the literature relating to endocrine dysfunction, including premature ovarian insufficiency (POI), in female PMCs, and then to consider whether endocrine dysfunction in itself may be predictive of other illnesses in female PMCs. A systematic review and pilot data from a semi-structured health questionnaire were used. Medline, Embase, and PsycInfo were searched for papers concerning PMCs and endocrine dysfunction. For the pilot study, self-reported diagnoses in females were compared between PMCs with endocrine dysfunction (*n* = 18), PMCs without endocrine dysfunction (*n* = 14), and individuals without the premutation (*n* = 15). Twenty-nine papers were identified in the review; the majority concerned POI and reduced fertility, which are consistently found to be more common in PMCs than controls. There was some evidence that thyroid dysfunction may occur more frequently in subgroups of PMCs and that those with endocrine difficulties have poorer health than those without. In the pilot study, PMCs with endocrine problems reported higher levels of fibromyalgia (*p* = 0.03), tremor (*p* = 0.03), headache (*p* = 0.01) and obsessive–compulsive disorder (*p* = 0.009) than either comparison group. Further larger scale research is warranted to determine whether female PMCs are at risk of endocrine disorders other than those associated with reproduction and whether endocrine dysfunction identifies a high-risk group for the presence of other health conditions.

## 1. Introduction

Humans typically have a CGG repeat length of under 45 repeats in the 5′ untranslated region of the fragile X mental retardation 1 (*FMR1*) gene. When the repeat length is expanded to between 55 and 200 repeats, an individual is classified as being an *FMR1* premutation carrier (PMC) [1]. CGG repeat lengths in the premutation range can expand further through maternal transmission; thus, female PMCs are at risk of having a child with over 200 CGG repeats. In individuals with repeat lengths of greater than 200, the fragile X mental retardation protein (FMRP) fails to be expressed and this results in the neurodevelopmental disorder known as fragile X syndrome. Aside from this increased risk of having an offspring with fragile X syndrome, being a female PMC was initially believed to have no mental or physical health implications. This view has since been disregarded and PMCs are now considered to be at heightened risk of developing health difficulties, most notably fragile X-associated tremor/ataxia syndrome (FXTAS) and fragile X premature ovarian insufficiency (FXPOI), but also potentially thyroid dysfunction, peripheral neuropathy, restless leg syndrome, fibromyalgia, migraines, hypertension, depression, anxiety, and obsessive–compulsive disorder (OCD) [2,3].

Much of the existing literature has focused on FXTAS; a neurological condition which typically presents from the 5th decade of life onwards [4]. Although FXTAS is primarily associated with tremor, ataxia, autonomic dysfunction, and cognitive decline [4,5], individuals with FXTAS have also been found to be at higher risk of other health problems including thyroid disease, hypotension, fibromyalgia, and possibly migraine, than those who do not have FXTAS [3,6,7]. The development of FXTAS is proposed to be linked to an overexpression of *FMR1* mRNA resulting in intranuclear inclusions, cell toxicity [8,9,10,11,12,13], and possible mitochondrial dysfunction [14,15]. The health problems co-occurring with FXTAS may be a direct consequence of FXTAS itself, or alternatively the presence of FXTAS may be an indicator of a more severe pathological process affecting multiple organ systems [4,6].

The prevalence of FXTAS is only about 8.3% in female PMCs which is considerably lower than in male PMCs (50% of 70–90 year olds) and may be attributed to the additional (unaffected) X chromosome within females having a protective factor [7,16]. The current research, therefore, may only provide a partial understanding of the health of female PMCs. In contrast, only females can have FXPOI, which is an endocrine condition characterised by absent or irregular periods, and menopausal symptoms (early menopause, hot flushes, and infertility); it has been identified that up to a quarter of PMCs show the most severe outcome of FXPOI—premature ovarian failure (POF), usually characterized as menopause before the age of 40 [17].

The pathology underlying FXPOI is still to be fully explained, but may be similar to that suggested for FXTAS. As both the brain and gonads are regions in the body where FMRP is highly expressed (and therefore *FMR1* highly transcribed) [10,18], cell toxicity could account for the heightened risk within female PMCs to develop both a neurological and a gonadal condition. Consistent with this, post-mortem studies of PMCs have documented the presence of intranuclear inclusions in non-central nervous system tissues, including, but not limited to, the gonads, thyroid, pituitary, and adrenal glands [19,20,21,22]. Endocrine disorders, such as premature ovarian insufficiency (POI) and thyroid dysfunction, may therefore represent markers of multi-system pathology in female PMCs, similar to the findings for FXTAS. Should this be the case then their presence would be an important clinical indicator for the need to thoroughly investigate affected individuals.

The intention of this study was therefore to review our existing knowledge around endocrine dysfunction and the *FMR1* premutation, and to consider whether or not the presence or absence of endocrine dysfunction is associated with markers of additional physical and/or mental illness. We initially carried out a systematic review of the literature relating to endocrine dysfunction in PMCs and then conducted a pilot study examining the self-reported health problems in female PMCs with endocrine dysfunction and compared these to PMCs without endocrine dysfunction and to non-carriers.

## 2. Materials and Methods

### 2.1. Literature Review

A systematic review was conducted to investigate the effect of endocrine problems in PMCs. All published articles indexed in Medline, Embase, and PsycInfo up to June 2016 were searched using the terms endocrine, FXPOI, ovar$, thyroid, menopause, pituitary, adrenal, hypothalamus, parathyroid, pineal, and pancreas; combined using an OR operator. The results were then combined using an AND operator with all studies which concerned human females and contained the terms fragile X or premutation. The reference lists of identified articles were also searched for articles meeting our inclusion criteria.

All abstracts were reviewed independently by two authors (A.M. and S.E.) and articles which were potentially suitable for inclusion were retrieved in full text and further reviewed by S.C. and A.S. To be included in the review, studies had to be published in a peer-reviewed journal and contain a statistical evaluation of endocrine dysfunction in a female PMC group (either within the group, or between PMCs and controls). Papers were excluded if they were a review, or based solely on understanding the molecular mechanisms underlying POI. Case studies and PMC prevalence studies within the broader population of patients with POI were also excluded.

### 2.2. Pilot Study

To further investigate the relationship between endocrine difficulties and health in female PMCs, a pilot study was conducted. Thirty-two female PMCs and fifteen non-carriers (NC) were recruited from the University of Edinburgh’s Patrick Wild Centre fragile X research registry. The fragile X research registry is an ongoing longitudinal study which aims to determine the clinical history of people with fragile X and the fragile X premutation. The nature of this registry means that the non-carriers were primarily drawn from families affected by fragile X syndrome. All participants completed a structured clinical history questionnaire. For 17 of the 32 PMCs the questionnaire was administrated through a face-to-face interview with a clinical psychologist or consultant psychiatrist. The remaining participants completed an online version. The online version of the questionnaire included additional questions, asking about the presence of conditions which had been repeatedly cited by participants during face to face interviews. Any item only listed within the online questionnaire is marked with an asterisk (*) within this methods section.

All participants, regardless of questionnaire version used, were asked about their personal demographics (age, gender, and *FMR1* status), whether they took medication or not, and to name the medication(s) used. Medications were subsequently grouped into one of eight categories (cardiovascular, digestive, metabolic, endocrine, neurological, mental health, analgesic, or other), which were confirmed by a consultant psychiatrist and a research nurse.

Participants were then asked about the function of their endocrine system (in particular the presence of any one of: early menopause, absence of or irregular menses, or hypo-/hyperthyroidism). The participants were also asked specific questions relating to dysfunction of other bodily systems. The body systems asked about included cardiovascular (heart and circulatory problems*), digestive (gastric difficulties), immune (allergies), musculoskeletal (hypermobility), sensory (hearing and vision difficulties not corrected by glasses), neurological (epilepsy/seizures, migraines/headaches, pins and needles/paresthesia* and FXTAS/tremor), and mental health difficulties (depression, anxiety disorders, and obsessive compulsive disorders). They were also asked about other pain-associated conditions (chronic pain*, arthritis*, endometriosis*, and fibromyalgia). For each condition the participant could reply yes (if they had been officially diagnosed), possibly/unsure (if they felt the diagnosis applied to them but had not received a formal diagnosis), or no (if the diagnosis did not apply to them). As this was a pilot, it was deemed more important to eliminate under-reporting bias than over-reporting, therefore, yes and maybe responses were combined together for coding. Participants only had to report the possible presence of one condition within a given body system, to be coded as having a potential difficulty within that body system.

All participants were asked if they experienced any other additional health problems, and these were manually assigned by the research team to the relevant body system classification. Of note, muscular pain was coded under pain-associated difficulties as opposed to musculoskeletal difficulties and a new coding, respiratory, was created to incorporate any lung-related difficulties. Any unassigned conditions remained categorized as ‘other’. The research team then counted the number of health difficulties (excluding endocrine issues) reported by each participant; this was used as a measure of overall health.

IBM SPSS Statistics for Windows, Version 22.0 (IBM Corp., Armonk, NY, USA) was used for data input and analysis. The PMCs were divided into two groups; the first group endorsed one or more of early menopause, irregular menses, or thyroid difficulty, and were therefore classed as having potential evidence of endocrine dysfunction; the second group did not endorse any of these conditions. The non-carrier (NC) control group did not have fragile X syndrome or carry the premutation. The groups were compared for age differences using a Kruskal-Wallis test. The significance level for all analyses was set at *p* < 0.05.

Fisher’s exact tests were used to compare the groups in terms of how many individuals were on medication (2-sided) and levels of polypharmacy (as defined by taking four or more medications). Fisher’s exact tests were also used to investigate group differences in each of the eight categories of medication (cardiovascular, digestive, metabolic, endocrine, neurological, mental health, analgesic, and other).

To ascertain if the endocrine group had reported significantly more health issues than the other two groups, a Fisher’s exact test was used. The groups were then compared for differences in reported health difficulties within each of the seven body systems (cardiovascular, digestive, immune, muscular/skeletal, respiratory, sensory, and neurological), mental health, and pain-associated conditions using Fisher’s exact tests. When significant differences (*p* < 0.05) between the groups were found for these categories, or results were approaching significance (*p* < 0.1), the specific conditions reported under each category were then compared between the groups using Fisher’s exact tests.

## 3. Results

### 3.1. Literature Review

**Figure 1:**
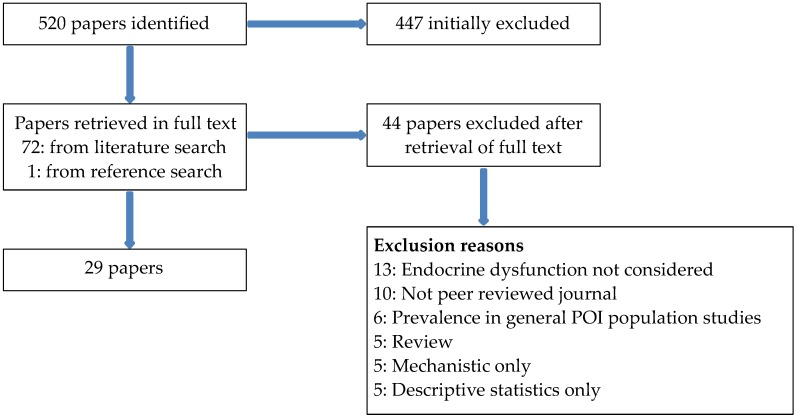
Summary of extraction process. POI: premature ovarian insufficiency.

A summary of the extraction process is given in Figure 1. Twenty-nine papers were identified that met the inclusion/exclusion criteria.

#### 3.1.1. Reproductive System

Table 1 summarises the studies which have considered reproductive difficulties in PMCs.

##### Primary Ovarian Failure/Primary Ovarian Insufficiency

Primary ovarian failure (POF)/POI are the most commonly investigated endocrine abnormalities in female PMCs. Studies have consistently shown female PMCs to be at significantly higher risk of experiencing POI than controls [23,24,25]. Female PMCs have an elevated risk of having an early menopause compared to both females with fragile X syndrome [17,26] and NC [17,24,25,27,28,29,30].

##### Premenopausal Ovarian Dysfunction

Evidence of ovarian dysfunction has also been identified in premenopausal PMCs, including shorter and more irregular menstrual cycles [3,23,27,45] and aberrant ovarian hormone levels. Follicular stimulating hormone (FSH) has been shown to be increased [36,39,45], with one study suggesting that this is mainly the case for PMCs between 30 and 39 years of age [24]; the latter study also suggested that anti-müllerian hormone (AMH) may be a more sensitive measure of ovarian dysfunction in PMCs as significant differences in AMH existed across all age groups. Differences in oestradiol have not been identified [36,45] while the levels of inhibin A and B may be reduced, but only in certain phases of the menstrual cycle [45]. As might be expected, the later development of POI has been linked to increased rates of uptake of fertility assistance, which are higher among PMCs with POI, compared to those without POI [46]. PMCs have also been observed to show a reduced response to ovarian hyperstimulation compared to the typical population [32].

##### Relationship with Genetic Characteristics

Several studies have focused on determining whether CGG repeat length was a risk factor in developing endocrine related health difficulties, and the focus of these studies has predominately been on menopause. Initial studies failed to find a significant correlation between CGG repeat length and the age of menopause [38]. However, one study found that the age of menopause only became associated with repeat size when individuals with high repeat numbers were excluded (over 100 repeats), and proposed that there was a curvilinear association between CGG repeat length and the age of menopause with the greatest risk occurring for those with medium length repeats [24]. This has since been confirmed by a number of further studies [29,33,37,44] and also extended to include premenopausal menstrual dysfunction [23]. The exact repeat length of greatest risk is not clear, with some studies suggesting that it is in the region of 80–100 repeats [23], whereas others finding that it may be slightly lower [33] or higher [37,44] than this.

The pattern of X chromosome inactivation (XCI) and the parental origin of the premutation have also been considered as potential risk factors for developing POI. One study has shown a significant relationship between paternally inherited mutations and early menopause/POI [35], while others have not found such a relationship [24,25,42]. No compelling evidence has been found for a relationship between skewed XCI in the development of POI [24,37,40,42,44].

#### 3.1.2. Other Endocrine Disorders

Table 2 summarises the studies which have considered other endocrine disorders in female PMCs.

The most commonly investigated endocrine disorders in female PMCs, other than POI, are thyroid disease and type II diabetes.

Several studies have reported that female PMCs do not show any increase in thyroid dysfunction although non-significant increases were generally identified [6,30,34]. Hunter et al. [6] did find that the presence of irregular menstruation is significantly associated with the presence of thyroid dysfunction. Coffey et al. [7] have identified that female PMCs with FXTAS had significantly higher rates of thyroid problems than controls (50% vs. 15.4%). Within this study, two thirds (*n* = 6) had hypothyroidism of an unspecified etiology and one third had hyperthyroidism. Winarni et al. [48] reported similar increases when considering autoimmune thyroid disease in PMCs with FXTAS. It is also important to note that in several studies the incidence of thyroid disease reported in the control populations was higher than the expected population prevalence, which may have minimised differences [34,47].

With regard to diabetes, no study has identified a significant difference in the prevalence of diabetes or pre-diabetes between female PMCs and controls [6,7,30,34].

One study has considered other markers of endocrine dysfunction, specifically pituitary or adrenal dysfunction, and salivary cortisol levels [34]. No significant differences were found between the groups, although it is worth noting that no cases of pituitary or adrenal dysfunction were found in either the PMCs or controls.

#### 3.1.3. Association between Endocrine Dysfunction and Other Health Conditions

Table 3 summarises those studies which have considered the relationship between endocrine dysfunction and health difficulties in female PMCs.

The available research only considers the association between POI or early menopause and non-reproductive health difficulties experienced by PMCs, i.e., there are no studies to date which have considered whether those with other endocrine disorders are at greater risk of health problems. Wheeler et al. [46] found that PMCs with POI were at increased risk of muscle weakness, dizziness, and nausea. Winarni et al. [47] found that compared to those without POI and to controls, PMC with POI have higher total rates of any immune-mediated disorders (defined in this study as including autoimmune thyroid disease, fibromyalgia, irritable bowel syndrome, Raynaud’s phenomenon, rheumatoid arthritis, Sjögren syndrome, lupus, multiple sclerosis, and optic neuritis). Three studies have investigated the effects of ovarian dysfunction on anxiety and depression and none has reported significant relationships [6,48,49], although Roberts et al. found a trend towards a significant relationship between POI and depression [49].

### 3.2. Pilot Study

There were no significant differences in mean age between the groups: endocrine PMC (*n* = 18; 47.7 years, standard deviation (SD) = 13.6), non-endocrine PMC (*n* = 14; 44.4 years, SD = 11.5), and the controls (*n* = 15; 42.0 years, SD = 11.5) (*p* = 0.33); 44% of the endocrine group, 50% of the non-endocrine PMC group, and all of the controls completed the online version of the questionnaire.

A summary of the results is given in Table 4. The endocrine PMC group reported having significantly more health problems overall than both the non-endocrine PMC and the control groups (*p* = 0.040) (Table 3). They were over three times more likely (44.4%) to report experiencing four or more health problems than controls (13.3%) and over six times more likely than PMCs without endocrine difficulties (7.1%).

PMCs with endocrine difficulties were significantly more likely to report a neurological issue than either the PMC non-endocrine or the control group (72.2% vs. 28.6% vs. 46.7%, respectively, *p* = 0.049, Table 4). Analysis of the sub-type of neurological issues reported revealed that only the endocrine group reported experiencing tremor (22.2%; *p* = 0.03) and that the rate of headache/migraine for both the endocrine (55.6%) and NC (46.7%) was higher than for PMCs without endocrine problems (7.1%) (*p* = 0.01; Table 4).

No significant differences were found in overall mental health although there was a non-significant trend towards a difference between the groups (*p* = 0.06; Table 4). The groups were however significantly different in their reporting of having a diagnosis of OCD. The PMC endocrine group (44.4%) was significantly more likely to report having symptoms than either of the other two groups (0% and 23.1%; *p* = 0.009). There was also a weak trend towards a significant difference in reported anxiety/depression (*p* = 0.097).

A higher number of PMCs in the endocrine group reported experiencing pain-associated conditions (55.6%, *p* = 0.045; Table 3) than the controls (14.3% and 26.7%). This was also reflected in medication use, with greater use of analgesic medication reported by endocrine PMCs (29.4%) compared to the non-endocrine PMCs (7.1%) and the controls (0%) (*p* = 0.04). When the individual pain-related conditions were investigated, fibromyalgia was only reported by the PMCs with endocrine difficulties (22.2%; *p* = 0.03), whereas no significant differences were found in the reported rate of arthritis between groups.

## 4. Discussion

### 4.1. Current Findings

We have identified that female PMCs with co-existing endocrine dysfunction are more likely to report having multiple health difficulties than those without endocrine dysfunction and non-carrier controls. Specifically, we identified clearly increased rates of fibromyalgia, headache, and tremor, as well as some evidence of increased levels of mental health difficulties. Analgesic use was also reported to be increased in PMCs with endocrine dysfunction.

The association between endocrine dysfunction and tremor is consistent with earlier corollary findings of abnormal endocrine tissue in PMCs with FXTAS [19,22]. Similarly, we also identified increased rates of fibromyalgia in PMCs with endocrine dysfunction, but not in those who do not show endocrine dysfunction. Although Winarni et al. [47] did not find such a relationship, increased rates of fibromyalgia have previously been reported in female PMCs more generally [7]. It has been suggested that this may result from increased levels of *FMR1* mRNA leading to alterations in pain neurotransmission in female PMCs [50]. It is possible, therefore, that endocrine dysfunction is an early marker of future FXTAS and of peripheral nervous system damage, manifesting as fibromyalgia. This altered neurotransmission may also be responsible for the increased levels of headache that we report, although it has also been suggested previously that there may be a more direct link between mitochondrial dysfunction and a susceptibility to migraines in PMCs [18]. Regardless, our data suggests that the identification of endocrine dysfunction in PMCs should prompt detailed assessment of neurological symptoms, including headache, and rheumatological pain.

Somewhat in contrast to Hunter et al. [6] and Kenna et al. [48] we also identified some evidence of increased levels of mental health difficulties, particularly when comparing the PMC groups with and without endocrine dysfunction. These differences were less apparent when the endocrine PMCs were compared to controls, although it is notable the control group in our study reported very high rates of mental health difficulties which may have obscured greater differences between the groups. It is also important to note that the age of participants may have obscured differences in our pilot study as depressive and anxiety disorders had been found to have a later onset in PMCs compared to the general population [51]. The high rates of OCD which we report in those with endocrine dysfunction have previously been identified in female PMCs [52] and are reported to be associated with increased levels of *FMR1* mRNA in PMCs more generally [53], but no other study has considered whether they relate specifically to endocrine dysfunction.

The current study is unable to address the mechanism accounting for the association between endocrine dysfunction in female PMCs and ill health that we report. It is possible that the *FMR1* premutation is associated with a multi-system pathological process, with the same or similar mechanisms occurring in different organ systems. Previous studies identifying intranuclear inclusions across multiple systems are in keeping with this theory [19,20,21,22]. Alternatively, it is possible that the health problems faced by PMCs are mediated by endocrine dysfunction and are not a direct effect of the premutation. To determine which of these is the case, future work should include an additional comparator group consisting of non-carriers with endocrine dysfunction to establish whether PMCs have a particular illness profile.

### 4.2. Limitations of Existing Research

The primary limitation of the existing research, including our own pilot, is the reliance on self-reported measures. These have provided useful insights and a foundation for further study but they are also open to recall and reporting bias. Reporting of an illness may also be affected by medication use, either increasing the likelihood that a condition is recalled and therefore reported, or decreasing the symptoms of a condition leading to under-reporting. As Hunter et al. [29] highlighted, this may be particularly true for menopausal symptoms as these are likely to have occurred many years before and may be masked by hormone replacement or birth control medications. Interestingly, Hunter et al. [29] also used both self-report of symptoms in combination with clinical examination and standardized scales and found certain symptoms to be under-reported while others were over-reported compared to the more standardised measures. Future research should employ standardised measures of data collection, including the use of health and prescription records to minimise bias.

Much of the existing endocrine-related research in PMCs has focused upon reproductive difficulties, with only a few studies examining diabetes and thyroid dysfunction and none investigating other endocrine disorders. Similarly, the relationship between endocrine dysfunction and other health conditions is relatively under-investigated, particularly compared to the large body of literature pertaining to the comorbidities associated with FXTAS. *FMR1* mRNA is widely transcribed in many tissues throughout the body and as such one would predict that dysfunction of other bodily systems may be associated with endocrine dysfunction.

Similarly, although our pilot data showed some clear results, it was also constrained by the limitations evident in the rest of the literature. In addition, as it was a pilot study, the sample size was small and we did not correct for multiple comparisons, meaning that significant associations may have been missed or overestimated. The small nature of the sample meant that we were unable to separate non-carriers into those with and without endocrine problems, meaning that this may have minimized differences between the groups. The questionnaire was administered in two slightly different formats, which may also have introduced bias. Furthermore, we did not include specific questions around the use of fertility assistance or difficulties conceiving. Our control group had high levels of mental health disorders in particular which may have obscured some results; this group was likely to consist of parents and family members of individuals with fragile X and therefore may have had higher levels of parenting stress than a population control group. Finally, we did not collect biological variables, such as CGG repeat number or *FMR1* mRNA levels, and are therefore unable to relate these to morbidity.

## 5. Conclusions

This paper has highlighted that *FMR1* PMCs with endocrine issues appear to be at significant risk of wider health difficulties, compared to those without evidence of endocrine dysfunction. They therefore represent a group in which detailed physical and mental health examination should be conducted. Future studies with large populations of female PMCs, using comprehensive clinical and biochemical examination and/or the use of health records, as opposed to solely self-report measures are required. The inclusion of a non-carrier group matched for endocrine dysfunction would be important in future research to determine whether the high rates of comorbid illness that we report in PMC with endocrine dysfunction are primarily related to PMC status per se, or are a secondary consequence of endocrine dysfunction regardless of cause. Potential confounding factors also need to be taken into account, such as the presence of a child with fragile X syndrome in the family. Further research at multiple levels of investigation from cellular to health behavior is strongly recommended to understand the profile of these females and inform genetic counselling, medical assessment and intervention.

## Figures and Tables

**Table 1:** Summary of studies considering reproductive health in female premutation carriers (PMCs).

Paper	Groups	*n*	Mean Age	Features Studied	Findings
Allen et al., 2007 [23]	PMC—low repeats PMC—mid repeats PMC—high repeats NC	127 237 70 521	42.7 (14.3) 45.8 (12.0) 35.8 (13.6) 56.1 (9.4)	POI Menstrual cycle Fertility Obstetric features	All PMC repeat lengths significantly associated with earlier premature ovarian failure (mid > high > low) Low and middle sized repeats show shorter and more skipped menstrual cycles. Mid-sized repeats showed irregular menstrual cycles. Low repeat lengths show early menarche. Mid-sized repeats associated with reduced fertility. Mid-sized repeats associated with increased DZ twinning. NSD in spontaneous abortion rates.
Allingham Hawkins et al., 1999 [17]	PMC FXS NC	395 128 237	-	Early menopause	Increased in PMC
Chonchaiya et al., 2010 [31]	PMC w parent w FXTAS NC	110 43	44.8 (8.2) 43.8 (8.1)	POI Menopausal symptoms Infertility	NSD Increased in PMC NSD
Coffey et al., 2008 [7]	PMC w FXTAS PMC w/o FXTAS NC	15 63 36	- - -	POI	NSD regardless of FXTAS status
Elizur et al., 2014 [32]	PMC NC	21 15	31.5 (3.4) 30.8 (4.3)	Response to controlled ovarian hyperstimulation	PMC had higher FSH:LH and gonadotrophin dosage and fewer retrieved oocytes. Number of retrieved oocytes showed non-linear association with repeat number and negative correlation with granulosa cell *FMR1* mRNA in PMCs.
Ennis et al., 2006 [33]	PMC	45	-	Early menopause	Curvilinear association with CGG number
Hall et al., 2016 [34]	PMC NC	33 13	54.2 (16.8) 47.0 (10.1)	Ovarian dysfunction	NSD
Hundscheid et al., 2000 [35]	PMC (paternal origin) PMC (maternal origin)	82 27	- -	Early menopause/POI	Earlier/increased in PMC with paternally inherited mutation
Hundscheid et al., 2001 [36]	PMC not on OCP NC/FXS not on OCP PMC on OCP NC/FXS on OCP	17 28 13 21	36 (2.2) 34 (4.7) 34 (5.3) 31 (4.5)	FSH Inhibin B 17β oestradiol	Higher in PMC regardless of contraceptive use NSD NSD
Hundscheid et al., 2003 [30]	PMC NC	152 112	52.7 (13.5) 41.1 (12.5)	Early menopause/POI Obstetric features	Increased in PMC NSD in number of pregnancies, children, spont. abortion, fetal loss or twinning
Hunter et al., 2008 [29]	PMC—low repeats PMC—mid repeats PMC—high repeats NC	134 248 78 541	49.1 (15.9) 43.3 (12.6) 38.8 (11.7) 42.4 (15.9)	Early menopause	Compared to NC, rates of early menopause highest in with mid-sized repeats (4x) then high and low sized repeats (2x). Significant genetic component to age of menopause even after controlling for PMC status.
Mailick et al., 2014 [37]	Postmenopausal PMC	88	-	Age at menopause	Curvilinear association between age at menopause and repeat length. NS relationship between menopause age and X inactivation ratio.
Mallolas et al., 2001 [38]	PMC w POI/early menopause PMC w/o POI/early menopause	21 206	- -	Parental origin of mutation	NSD
Murray et al. 1999 [39]	PMC NC/FXS	19 32		FSH	Increased FSH levels in PMC group
Murray et al., 2000 [28]	PMC NC/FXS	116 236	-	Early menopause Obstetric features Inhibin	Menopause occurs at significantly younger age in PMC. Repeat size and skewed X inactivation not related to age of menopause. Subset showed NSD in twinning or unfavourable pregnancy outcome. No relationship between repeat size and inhibin concentrations in PMC.
Rodriguez-Revenga et al., 2009 [40]	PMC w POI PMC w/o POI NC	40 220 220	-	POI	No relationship between skewed X-inactivation and POI.
Rohr et al., 2008 [41]	PMC < 70 repeats PMC > 70 repeats	- -	18–50 18–50	FSH AMH	PMC with >70 repeats had higher FSH levels than those with <70 repeats in 31–40 year old age group PMC with >70 repeats had higher AMH levels than those with <70 repeats in all age groups tested
Schwartz et al., 1994 [27]	PMC FXS NC (relatives) NC (non-relatives)	92 39 109 50	46 33 37 33	Menstrual cycle Early menopause Gynaecological complications Spontaneous abortions	More irregular in PMC Increased in PMC Increased in PMC compared to related NCs NSD
Spath et al., 2010 [42]	PMC w POI PMC w/o POI NC w POI	37 64 25	45.9 (13.2) 47.1 (12.7) 31.7 (19.5)	POI	No relationship between skewed X-inactivation and POI
Spath et al., 2011 [43]	PMC NC	517 551	51.5 53.5	Age at menopause	Within PMC, age at menopause significantly predicted using multivariate analysis by CGG repeat length, mean menopausal age of first degree relatives and smoking. CGG repeat length showed non-linear relationship with risk increased between 62 and 120 repeats.
Sullivan et al., 2005 [24] *	PMC NC	183 324	44.3 (13.5) 42.3 (14.6)	POI/early menopause FSH	PMC at increased risk of POI and early menopause compared to NC; highest rates with mid-sized repeat lengths; no association between POI/early menopause and parental origin. Subgroup showed no increase in FSH in PMC but further sub-analysis showed increased levels in PMC aged 30–39 years old. No relationship between FSH and repeat length, X inactivation ratio or parental origin.
Tejada et al., 2008 [44]	PMC w POI PMC w/o POI	25 17	- -	POI	NSD in mRNA, X inactivation ratio and CGG repeat lengths. POI associated most with >100 repeats.
Vianna-Morgante et al., 1999 [25]	PMC FXS NC	101 37 55	43.3 (11.8) 36.6 (9.4) 40.4 (12.7)	Early menopause/POI	PMC at increased risk of early menopause compared to FXS and NC. No relationship between POI and parental origin.
Welt et al., 2004 [45]	PMC NC	11 22	34.5 (5.7) 34.6 (5.8)	Menstrual cycle FSH LH Inhibin A Inhibin B Oestradiol Progesterone	Reduced total cycle length in PMC driven by reduced follicular phase Increased in PMC throughout follicular and luteal phases NSD Decreased in PMC in early and mid-luteal phases Decreased in PMC in early and mid-follicular and early luteal phases NSD Decreased in PMC in follicular phase
Wheeler et al., 2014 [46]	PMC w POI PMC w/o POI	73 365	48.6 (11.7) 48.9 (12.2)	Menstrual cycle Fertility Obstetric features	POI associated with absent or irregular menstrual periods POI associated with greater use reproductive assistance/fertility drugs POI associated with lower rates of precipitous labour (under 2 h)

Age is given as mean (standard deviation); PMC, premutation carriers; NC, non-carriers; FXS, fragile X syndrome; FXTAS, fragile X associated tremor ataxia syndrome; POI, premature ovarian insufficiency; NSD, no significant difference; FSH, follicular stimulating hormone; LH, luteinizing hormone; OCP, oral contraceptive pill; AMH, anti-müllerian hormone; MZ, monozygotic; DZ, dizygotic.

**Table 2:** Summary of studies considering non-reproductive endocrine issues in PMCs.

Paper	Groups	*n*	Mean Age	Features Studied	Findings
Chonchaiya et al., 2010 [31]	PMC w parent w FXTAS NC	110 43	44.8 (8.2) 43.8 (8.1)	Thyroid disease Diabetes	NSD NSD
Coffey et al., 2008 [7]	PMC w FXTAS PMC w/o FXTAS NC	18 127 69	59.2 (10.3) 42.3 (11.5) 45.8 (14.9)	Thyroid disease Type II diabetes	Increased only in PMC w FXTAS when compared to subset of age matched controls (57.1 years old) NSD
Hall et al., 2016 [34]	PMC NC	33 13	54.2 (16.8) 47.0 (10.1)	Thyroid disease Pituitary/adrenal dysfunction Thyroid antibodies Low 8 a.m. cortisol Prediabetes	NSD None detected in either group NSD NSD NSD
Hundscheid et al., 2003 [30]	PMC NC	152 112	52.7 (13.5) 41.1 (12.5)	Thyroid disease Type II diabetes	NSD NSD
Hunter et al., 2010 [6]	PMC NC	203 334	37.1 (8.4) 32.5 (10.1)	Thyroid disease Type II diabetes	NSD. Presence of irregular menstruation significantly associated with presence of thyroid problems NSD
Winarni et al. (2012) [47]	PMC w FXTAS PMC w/o FXTAS NC PMC w FXTAS PMC w/o FXTAS NC PMC w POI PMC w/o POI NC	56 288 72 45 158 57 41 147 50	- - - >40 >40 >40 >40 >40 >40	Autoimmune thyroid disease	PMC w FXTAS > PMC w/o FXTAS = NC PMC w FXTAS > PMC w/o FXTAS = NC NSD

Age is given as mean (standard deviation).

**Table 3:** Summary of studies considering co-occurring health difficulties in female PMCs with endocrine dysfunction.

Paper	Groups	*n*	Mean Age	Features studied	Findings
Hunter et al., 2010 [6]	PMC NC	203 334	37.1 (8.4) 32.5 (10.1)	Anxiety Depression	Ovarian reserve did not predict either depression or anxiety in PMCs
Kenna et al., 2013 [48]	Premenopausal PMC Perimenopausal PMC Postmenopausal PMC	17 7 22	46.2 (6.2) 43.7 (5.7) 50.1 (4.1)	Anxiety Depression	NSD between groups; no significant relationship between prevalence of anxiety or depression and age of menopause or postmenopausal use of HRT
Roberts et al., 2016 [49]	PMC w/POI PMC w/o POI	34 49	- -	Anxiety Depression	POI did not significantly predict anxiety POI showed trend towards significantly predicting depression
Wheeler et al., 2014 [46]	PMC w POI PMC w/o POI	73 365	48.9 (12.2) 48.6 (11.7)	Anxiety Depression ADHD Learning disability Speech/language dis. Hypertension Heart disease Diabetes Autoimmune disorder Thyroid disease GI problems Seizures Specific physical symptoms	NSD NSD NSD NSD NSD NSD NSD NSD NSD NSD NSD NSD PMC with POI reported significantly more muscle weakness, dizziness and nausea
Winarni et al. (2012) [47]	PMC w POI PMC w/o POI NC	41 147 50	>40 >40 >40	Immune mediated disorders	PMC w POI > PMC w/o POI > NC

Age is given as mean (standard deviation); GI, gastrointestinal; HRT, hormone replacement therapy.

**Table 4:** Physical and mental health issues in PMC.

Group		Endocrine		Non-endocrine				
Analysis		PMC		PMC		NC		*p*
		%	*n = 18*	%	*n = 14*	%	*n = 15*	
Medication								
Takes medication		61.1	18	50	14	28.6	14	0.20
Number of medications taken								
	0–3	72.2	18	92.9	14	85.7	14	
	3+	27.8		7.1		14.3		0.30
Type of medication used								
	Cardiovascular	0	17	7.2	14	7.1	14	0.52
	Digestive	38.9	18	21.4	14	13.3	15	0.26
	Metabolic	5.9	17	7.1	14	7.1	14	1.00
	Endocrine	17.6	18	0	14	0	15	0.10
	Neurological	11.8	18	14.3	14	0	15	0.44
	Mental health	29.4	18	21.4	14	14.3	14	0.61
	Analgesic	29.4	18	7.1	14	0	15	0.04 *
	Other	23.5	18	21.4	14	7.1	14	0.50
Health difficulties								
Number of health difficulties			18				15	
	0–3	55.6		92.9	14	86.7		
	4+	44.4		7.1	14	13.3		0.04 *
Cardiovascular		27.8	18	7.1	14	13.3	15	0.36
Digestive		38.9	18	21.4	14	13.3	15	0.26
Immune		22.2	18	21.4	14	26.7	15	1.00
Muscular/skeletal		27.8	18	7.1	14	20.0	15	0.38
Respiratory		5.6	18	14.3	14	0	15	0.38
Sensory		27.8	18	14.3	14	33.3	15	0.54
Neurological		72.2	18	28.6	14	46.7	15	0.049 *
	Tremor	22.2	18	0	14	0	15	0.03 *
	Headache	55.6	18	7.1	14	46.7	15	0.01 *
	Paraesthesia	22.2	18	0	14	6.7	15	0.17
	Seizures	5.6	18	23.1	13	0	15	0.09
Mental health		77.8	18	35.7	14	66.7	15	0.06
	Anxiety/depression	58.8	17	35.7	14	71.4	14	0.19
	OCD	44.4	18	0	14	23.1	13	0.009 *
Pain-associated conditions		55.6	18	14.3	14	26.7	15	0.045 *
	Arthritis	16.7	18	0	14	20.3	15	0.29
	Fibromyalgia	22.2	18	0	14	0	15	0.03*
	Endometriosis	0	18	0	14	0	15	N/A

* Significant at *p* < 0.05 level; *n* = number of participants who completed each section of the questionnaire. OCD, obsessive–compulsive disorder.
